# Supplementary material for: The Effect of Breed and Age on the Growth Performance, Carcass Traits and Metabolic Profile in Breast Muscle of Chinese Indigenous Chickens
Source: Foods. 2022 Feb 7;11(3):483. doi: 10.3390/foods11030483 (PMC8834177; doi:10.3390/foods11030483)
Supplement: Supplementary file 1 [file foods-11-00483-s001.zip › supplementary figures.pdf]

foods

MDPI

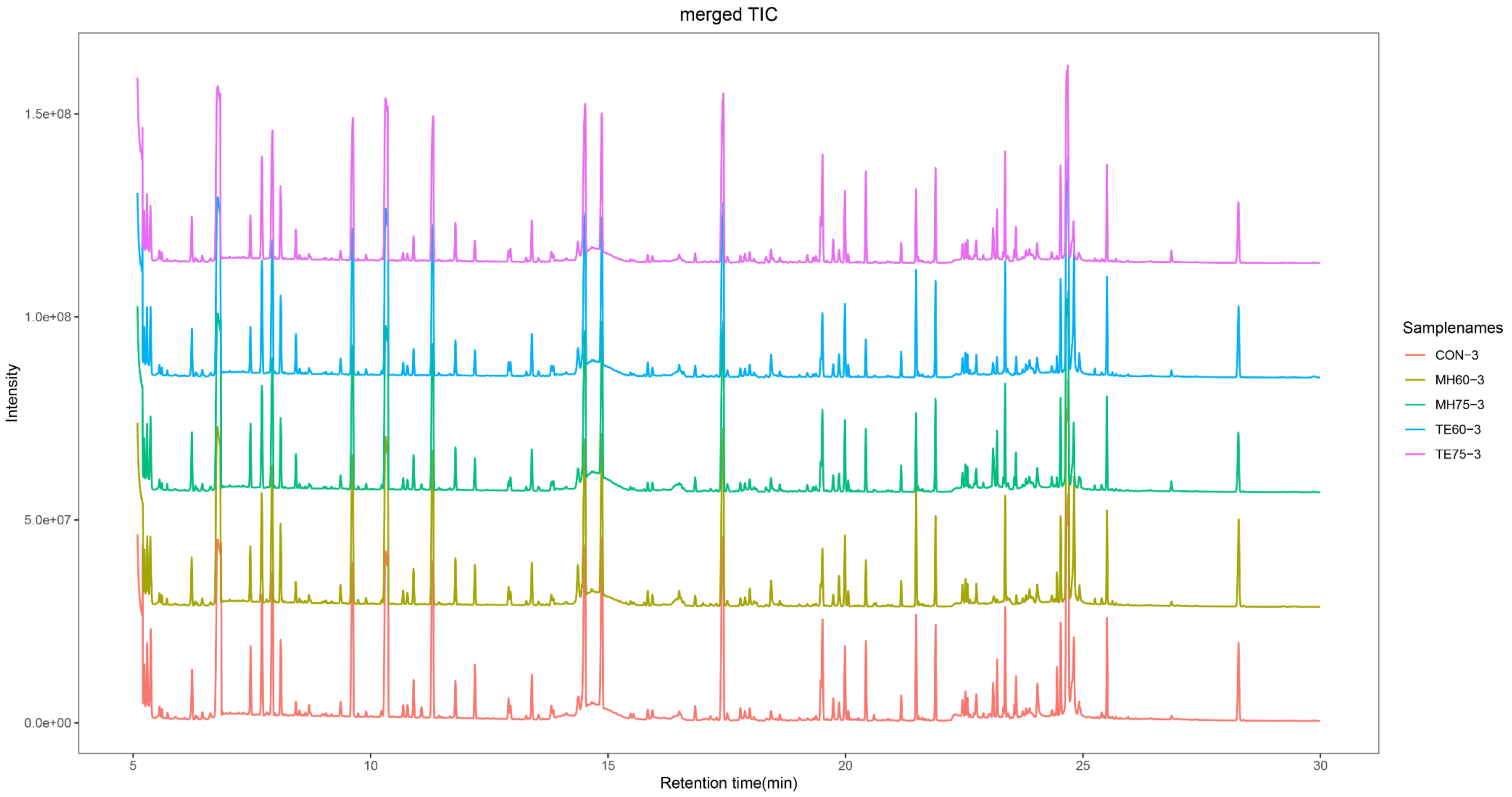

Figure S1. Representative GC-MS total ion chromatograms of samples from the CON, MH60, MH75, TE60 and TE75 groups.

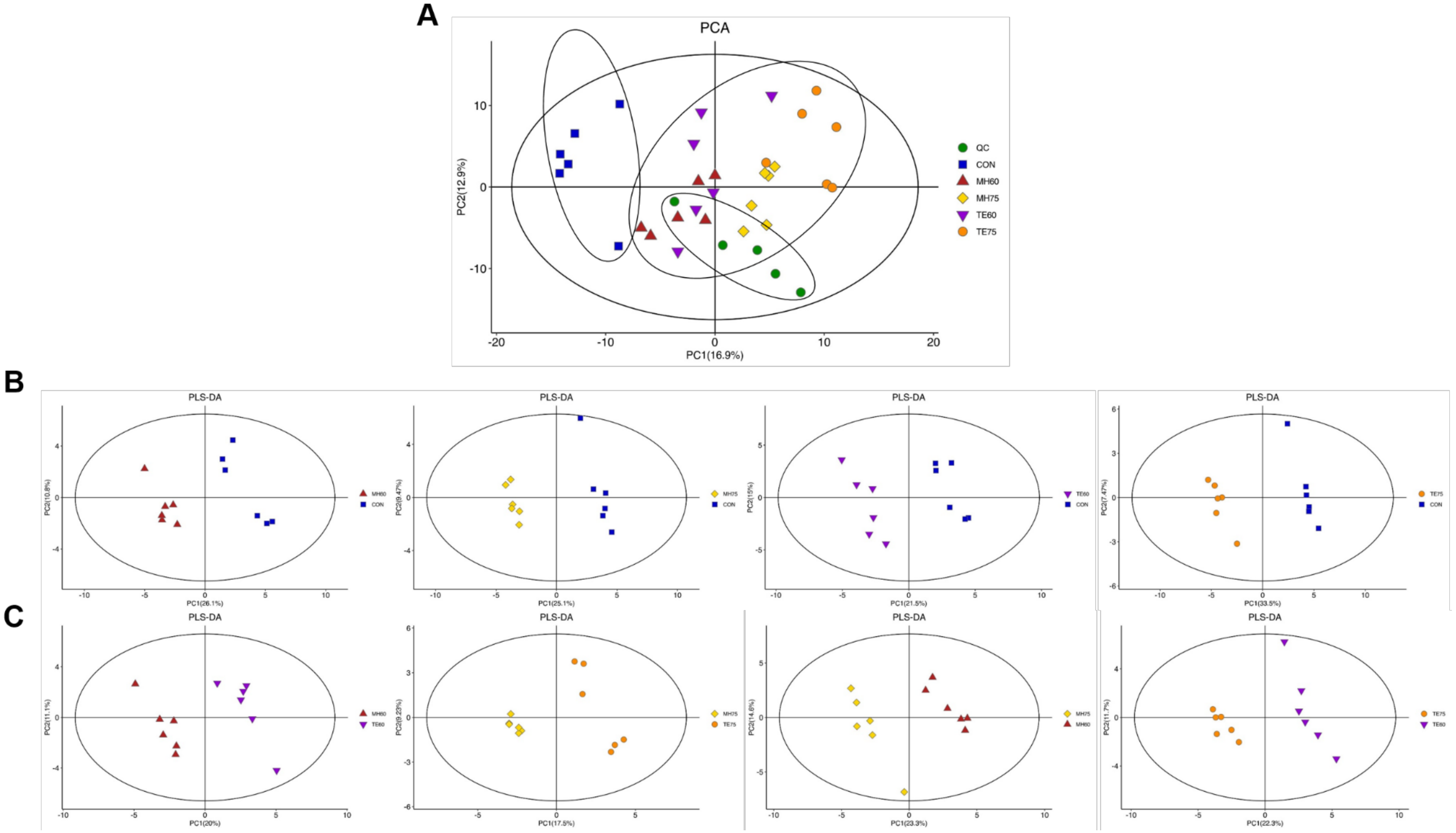

Figure S2. Principal component analysis (PCA) derived from the GC-MS profiles of muscle samples obtained from the five groups (A); projection to latent structure discriminant analysis (PLS-DA) score plots between the 817 crossbred broiler and the indigenous yellow-feathered chickens (B); . PLS-DA score plots between the MH60 group vs. the TE60 group, the MH75 group vs. the TE75 group, the MH75 group vs. the MH60 group, and the TE75 group vs. the TE60 group.

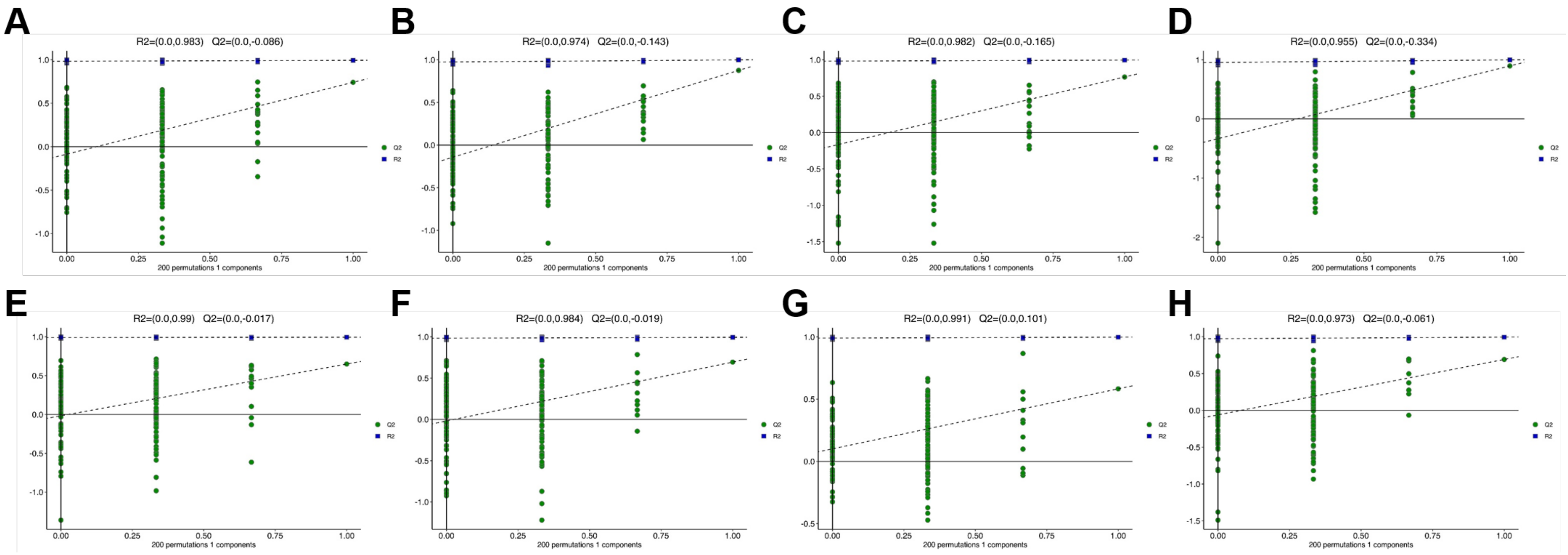

Figure S3. The validation plots of the permutation test for the OPLS-DA models derived from the GC-MS profiles of muscle samples obtained from the MH60 group vs. the CON group (A), the MH75 group vs. the CON group (B), the TE60 group vs. the CON group (C), the TE75 group vs. the CON group (D), the MH60 group vs. the TE60 group (E), the MH75 group vs. the TE75 group (F), the MH75 group vs. the MH60 group (G), and the TE75 group vs. the TE60 group (H).
